# A Cell-Based Metabonomics Approach to Investigate the Varied Influences of Chrysophanol-8-O-β-D-Glucoside With Different Concentrations on L-02 Cells

**DOI:** 10.3389/fphar.2018.01530

**Published:** 2019-01-09

**Authors:** Meichen Liu, Xiaohong Gong, Yunyun Quan, Yimeng Zhou, Yunxia Li, Cheng Peng

**Affiliations:** Pharmacy College, State Key Laboratory Breeding Base of Systematic Research, Development and Utilization of Chinese Medicine Resources, Chengdu University of Traditional Chinese Medicine, Chengdu, China

**Keywords:** chrysophanol-8-O-β-D-glucopyranoside, L-02 cell, metabonomics, high content analysis, amino acid metabolism

## Abstract

In this study, the effects of different concentrations of chrysophanol-8-O-β-D-glucoside (C-8-O-β-D-glu) on L-02 liver cells were analyzed by high content analysis (HCA) and metabonomics to explore the potential mechanism involved. The results showed that low concentrations (12 and 24 μM) of C-8-O-β-D-glu increased the cells viability significantly, while high concentration (96 μM) showed significant cytotoxicity on L-02 cells. HCA was applied to analyze the changes of nuclei and mitochondria after the cells being exposed to C-8-O-β-D-glu for 24 h. The results showed high concentration (96 μM) of C-8-O-β-D-glu significantly reduced the number of living cells, increased average nucleus area, DNA content and mitochondrial membrane potential (MMP). Then non-target metabonomics was carried out to identify potential biomarkers and metabolic pathways of L-02 cells impacted by C-8-O-β-D-glu. Eleven important potential biomarkers associated with four metabolic pathways were identified in this analysis. Dysregulation of alanine, aspartate and glutamate metabolism were observed in both LCG and HCG. In addition, low concentration (24 μM) of C-8-O-β-D-glu would impact arginine and proline metabolism. High concentration (96 μM) of C-8-O-β-D-glu would impact phenylalanine metabolism and beta-alanine metabolism. Alanine, aspartate and glutamate metabolism, arginine and proline metabolism, phenylalanine metabolism, beta-alanine metabolism were involved in different effects of C-8-O-β-D-glu on L-02 cells.

## Introduction

Anthraquinones are widely distributed in nature as the secondary metabolic products of various plants. Most anthraquinones are derivatives of tricyclic aromatic organic compound 9,10-anthracenedione (Duval et al., 2016) and can be classified into free anthraquinones and glycosylated anthraquinones according to chemical structure. Both them have been proved to possess a broad range of pharmacological effects such as protecting liver (Alisi et al., 2012), antioxidant (Brkanac et al., 2015; Zhao et al., 2016), anticancer (Chen et al., 2015; Cui et al., 2016), anti-inflammatory (Chen et al., 2016), antileukemic (Ghoneim et al., 2013), anticoagulant (Seo et al., 2012), anti-diabetic (Lee and Sohn, 2008), antibacterial (Chen and Zhu, 2013) and protecting kidney (Tan et al., 2004) etc.

However, anthraquinones are reported to induce liver damage (Yu et al., 2011; Wang et al., 2017) in recent years. Many researches have been carried out to investigate the hepatotoxicity of free anthraquinones, and have elucidated toxicity mechanisms involving inflammation, oxidative stress, mitochondrial dysfunction, the expression of metabolic enzymes and hepatocyte apoptosis (Lai et al., 2009; Qu et al., 2013). But the hepatotoxicity of glycosylated anthraquinones has not received much attention until now.

**FIGURE 1:**
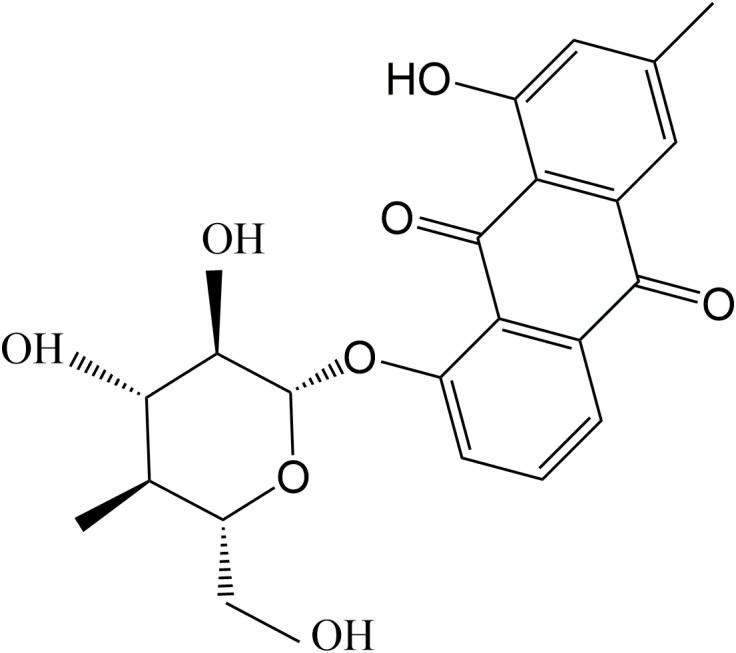
Chemical structure of Chrysophanol-8-O-β-D-glucopyranoside.

As one of the glycosylated anthraquinones, chrysophanol-8-O-β-D-glucoside (C-8-O-β-D-glu, Figure 1) exhibits various activities including anti-aging, neuroprotective (Chen et al., 2008), antiplatelet and anticoagulant activities (Seo et al., 2012). However, the effect of C-8-O-β-D-glu on liver has not been investigated. Pharmacokinetics study has proved C-8-O-β-D-glu could be absorbed into blood in intestine and transported to liver (Zhao et al., 2011). Therefore, C-8-O-β-D-glu might directly act on liver cells to affect the physiological state of liver which was verified in our laboratory.

In present study, HCA was applied on L-02 cell line to provide cell morphological information and quantify specific fluorescent targets, and metabonomics was used to qualitatively and quantitatively analyze the variations of differential metabolites and the disturbed metabolic pathways to further clarify the varied influences of C-8-O-β-D-glu with different concentrations on liver cells.

## Materials and Methods

### Chemicals and Drugs

Methanol (HPLC grade) was purchased from Wokai Chemical Technology Co., Ltd. (Shanghai, China). Acetonitrile (HPLC grade) was obtained from Merck Chemicals (Shanghai, China). Formic acid (HPLC grade) was purchased from TCI Chemical Industry Development Co., Ltd. (Shanghai, China). Other reagents and chemicals were of analytical grade and purchased from Kelong Chemical Reagent Factory (Chengdu, China).

C-8-O-β-D-glu (purity above 98%) was purchased from Chengdu Chroma-Biotechnology Co., Ltd. (Chengdu, China), and was dissolved in DMSO (MP Biomedicals LLC, France) at the concentration of 96 mM as stock solution.

### Cell Culture

The entire research was carried out on human liver cell line L-02 cells purchased from KeyGEN BioTECH (Nanjing, China). The cells were cultivated in DMEM medium (GIBCO, United States) supplemented with 10% FBS (GIBCO, United States) and antibiotics (100 U/ml of penicillin and 0.1 mg/ml of streptomycin). They were incubated at 37°C, 5% CO_2_ in LabLine CO_2_ Incubator (Thermo, United States) and subcultured every 2 days.

### C-8-O-β-D-Glu Stability Analysis

C-8-O-β-D-glu stock solution was diluted to 96 μM with cell culture medium. After being incubated for 0, 6, 12, 24, and 48 h, 10 μL sample were analyzed (*n* = 3) by HPLC. Agilent 1260 Infinity HPLC system (Agilent, United States) was applied to conduct the analysis on a Zorbax Eclipse Plus C_18_ column (4.6 × 250 mm, 5 μm, Agilent, United States) at 30°C. The analyte was eluted by 0.1% phosphoric acid water: methanol (20:80) at 1 ml/min for 10 min.

### MTT Assay

Exponentially growing cells were plated in 96-well plate (Costar, United States) at the density of 6 × 10^3^ per well and grew in incubator for 24 h. At the same time, the culture medium with 0.1% DMSO were added into wells without cells to zero the OD value. The adhered cells were treated with different concentrations of C-8-O-β-D-glu (0, 12, 24, 48, and 96 μM) prepared in DMEM medium supplemented with 0.1% DMSO and cultured for 24 h. Then the supernatants were carefully removed, and 20% 3-(4, 5-dimethylthiazol-2-yl) 2, 5- diphenyltetrazolium bromide (MTT) were added. After 4 h, MTT-formazan crystals were dissolved by 150 μL DMSO. The absorbance of the solution was measured at 570 nm (*n* = 6). The influence of different concentrations on cells viability was calculated by the percentage of viable cells between drug experimental groups and the CG.

### High Content Analysis

Exponentially growing cells were plated in 96-well plate at the density of 6 × 10^3^ per well and grew in incubator for 24 h. Then the cells were treated with different concentrations of C-8-O-β-D-glu (0, 24, 48, and 96 μM) prepared in DMEM medium supplemented with 0.1% DMSO for another 24 h. After that, the medium was removed and the cells were washed with PBS. Then cells were stained by 50 μL freshly prepared Rho123, 10 μM (Beyotime, China), per well. After 30 min incubation without light, the dye was removed. Cells were washed with PBS and then exposed to Bisbenzimide H 33342(10 μM, Sigma, United States) for 15 min in incubator for imagination.

Cells were imaged under High Content Screening ImageXpress^®^ Micro (Molecular Devices, United States). The detection conditions were set as follows: the first channel wavelength was 350 nm/460 nm irradiation for Bisbenzimide H 33342 labeled nuclei. The second channel wavelength was 507 nm/530 nm irradiation for Rho123 labeled mitochondria. Five images were captured per well for image analysis performed with MetaMorph image processing. Cells number was directly counted by the software. Average nucleus area, DNA content and MMP were calculated based on the data recorded.

Average nucleus area=wavelength 1 stained area/total cells

DNA content=wavelength 1 integrated intensity/total cells

MMP=wavelength 2 stained integrated intensity/total cells

### Metabonomics

#### Cell Sample Collection and Preparation

Exponentially growing L-02 cells were plated in 175 cm^2^ culture flasks (Costar, United States) at the density of 5 × 10^6^ per flask and grew in incubator for 24 h. After that, the drug experimental groups were treated with C-8-O-β-D-glu of different concentrations (24, 48, and 96 μM) for another 24 h. CG was treated with the culture medium with 0.1% DMSO as the same way. Then the supernatants were removed, and the flasks were washed with PBS twice. The cells were digested into suspension by 0.25% trypsin. 1 × 10^7^ cells were looked as one volume and five volume PBS (4°C) was added to resuspend the cells which was centrifuged at 200 *g* for 4 min. After repeating the process three times, the cells were quenched by liquid nitrogen after removing the supernatants.

The cells were resuspended in 500 μL methanol (-80°C) for 30 s. 60 μL of 0.2 mg/mL nonadecylic acid in methanol and 60 μL of 10 mM d4-alanine in methanol as internal quantitative standards were added into the cells. After 30 s vortex, the mixture was snap-frozen in liquid nitrogen. The frozen-quenched cells were thawed, vortexed for 30 s and centrifuged at 800 g for 1 min. The supernatant was transferred to a microcentrifuge tube on dry ice and the cell pellet was resuspended in methanol (-80°C). The above step was repeated and the cells were vortexed for 30 s and pelleted by centrifugation. The supernatant was pooled with the previous methanol fraction and the cell pellet was resuspended in 250 μL ice cold Milli-Q water. The freeze-thaw cycle was repeated for the last time and then the cells were vortexed for 30 s and pelleted by centrifugation at 15000 g for 1 min. The supernatant was removed and pooled with the previously pooled methanol extracts, and any remaining cell debris was removed by centrifugation at 15000 g for 1 min. The supernatant was removed to a fresh tube and blow-dried by vacuum concentration at 30°C. The samples were dissolved with 300 μL methanol aqueous solution (1:1, 4°C), and filtered by 0.22 μm membrane before UPLC – MS/MS detection.

Twenty microliter sample was collected from each sample to prepare quality control (QC) samples which were injected before and after the injection of the test samples.

#### Chromatography and Mass Spectrometry

Chromatographic separation was processed in a Thermo UHPLC Ultimate 3000 system equipped with an ACQUITY UPLC^®^ HSS T3 (150 × 2.1 mm, 1.8 μm, Waters) column at 40°C. The temperature of the autosampler was set at 4°C. Gradient elution of analytes was performed with 0.1% formic acid in water (A) and 0.1% formic acid in acetonitrile (B) at the flow rate of 0.25 mL/min: 0∼1 min, 2% B; 1∼9.5 min, 2∼50% B; 9.5∼14 min, 50∼98% B; 14∼15 min, 98% B; 15∼15.5 min, 98∼2% B; 15.5∼17 min, 2%. 3 μL sample was injected for analysis after equilibration.

ESI-MS^n^ experiments were executed on a Thermo Q Exactive Plus mass spectrometer with the spray voltage of 3.5 and -3.5 kV in positive and negative modes, respectively. Sheath gas and auxiliary gas were set at 30 and 10 arbitrary units, respectively. The capillary temperature was 325°C. The Orbitrap analyzer scanned over a mass range of m/z 70–1000 for full scan at a mass resolution of 70000. Data dependent acquisition (DDA) MS/MS experiments were performed with HCD scan. The normalized collision energy was 30 eV. Dynamic exclusion was implemented with an exclusive duration of 10 s.

#### Data Processing and Pattern Recognition Analysis

All raw UPLC-MS/MS data were converted into mzXML format (xcms input file format) by Proteowizard software (v3.0.8789) (Smith et al., 2006). The XCMS package of R (v3.3.2) was used for the peaks identification, peaks filtration and peaks alignment. The main parameters were bw = 5, ppm = 15, peakwidth = c(10, 120), mzwid = 0.015, mzdiff = 0.01, method = “centWave.” After that, the data matrix including mass to charge ratio (m/z), retention time and intensity information was derived and exported to excel for subsequent analysis. In order to compare the data of different magnitude, batch normalization of peak area was applied.

The data set of all samples, consisting of retention time and normalized peak area of metabolites, was imported into Simca-P v13.0 software (Umetrics AB, Umea, Sweden) and R language tools package for multivariate statistical analysis. The data was pre-processed by unit variance scaling and mean-centered method. Then, the data were processed by PCA, partial least squares-discriminant analysis (PLS-DA) and OPLS-DA to discriminate CG and drug experimental groups.

Variables with VIP exceeding 1 showed a higher influence on the classification. Therefore, the metabolite biomarkers were screened with VIP value (based on OPLS-DA) ≥1 and *q*-value (*p*-value corrected for multiple hypothesis testing by using FDR correction) ≤0.05.

### Metabolite Biomarker Identification and Metabolic Pathway Analysis

All metabolite biomarkers were identified based on exact molecular weight, retention time and MS/MS information at first. Then, several databases including Human Metabolome Database (HMDB^1^), Metlin^2^, massbank^3^, LipidMaps^4^, and mzclound^5^ were used for further confirmation. To further determine the metabolic patterns of differential metabolites in each group, the dataset was scaled by heat-map package in R(v3.3.2). The samples and metabolites were bidirectionally clustered. To determine the relevant metabolic pathways, the metabolic pathway analysis of potential biomarkers was performed using Kyoto Encyclopedia of Genes and Genomes (KEGG^6^) database. And possible metabolic pathways were identified by metabolic pathways enrichment and topology analysis through MetPA^7^ database.

### Statistical Analysis

Data were analyzed by SPSS 21.0 for Windows (SPSS Inc.). Results were represented as mean ± SD and evaluated using the two-tailed unpaired student’s *t*-test or one-way analysis of variance. The *p* < 0.05 was considered to be significant and *p* < 0.01 to be very significant.

## Results

### C-8-O-β-D-Glu Stability Analysis

The stability of C-8-O-β-D-glu (96 μM) in cell culture medium was analyzed within 48 h. The concentration of C-8-O-β-D-glu incubated for 0, 6, 12, 24, and 48 h were shown in Table 1. The RSD (<2%) of drug concentration at each time point indicated that C-8-O-β-D-glu was stable in cell culture medium which guaranteed the study was focused on C-8-O-β-D-glu instead of other chemicals.

**Table 1:** Contents of C-8-O-β-D-glu in cell culture medium after being incubated for 0, 6, 12, 24, and 48 h. Each value represented the mean ± SD (*n* = 3).

Time (h)	0	6	12	24	48
Content (μM)	95.25 ± 0.38	91.43 ± 2.28	94.08 ± 1.49	92.95 ± 1.77	93.19 ± 1.39
RSD	1.5%				


### MTT Assay

**FIGURE 2:**
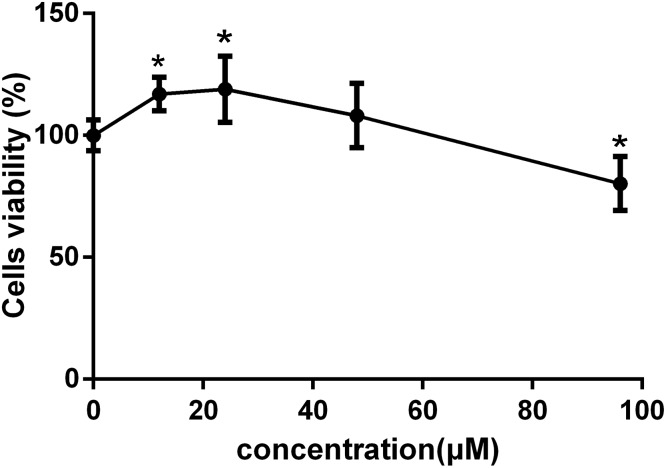
The viability of L-02 cells affected by different concentrations of C-8-O-β-D-glu determined by MTT assay. Each value represented the mean ± SD (*n* = 6). One-way analysis of variance (ANOVA) was used to calculate significant difference. ^∗^*P* < 0.05, compared with the CG.

To investigate the effects of C-8-O-β-D-glu on L-02 cells viability and select the concentrations for the follow-up studies, MTT assay was performed at first. In CG, the same FBS and antibiotics were added to eliminate the blank interference. As shown in Figure 2, 12 and 24 μM C-8-O-β-D-glu significantly increased the cells viability (*p* < 0.05), while 48 μM exert no influence. However, when concentration increased to 96 μM, significant inhibition of the viability of L-02 cells after 24 h was observed (*p* < 0.05). It indicated that lower concentrations of C-8-O-β-D-glu increased cells viability, but high concentration led to liver injury. Consequently, 24, 48, and 96 μM were selected as the low, medium and high concentrations of C-8-O-β-D-glu, respectively in the follow-up studies.

### High Content Analysis

**FIGURE 3:**
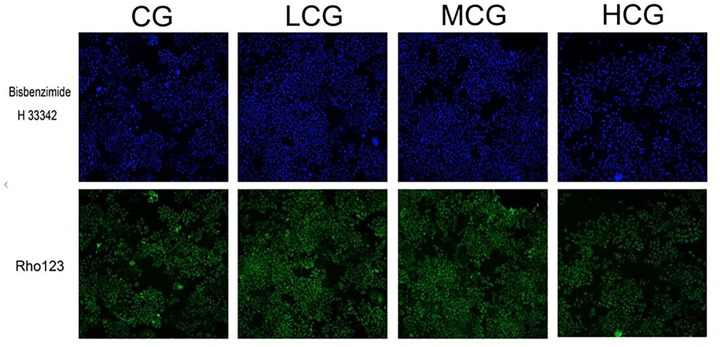
The representative cell staining images of the four groups. Blue fluorescence indicated that the nuclei were stained by Bisbenzimide H 33342. Green fluorescence indicated that mitochondria were stained by Rho123.

**FIGURE 4:**
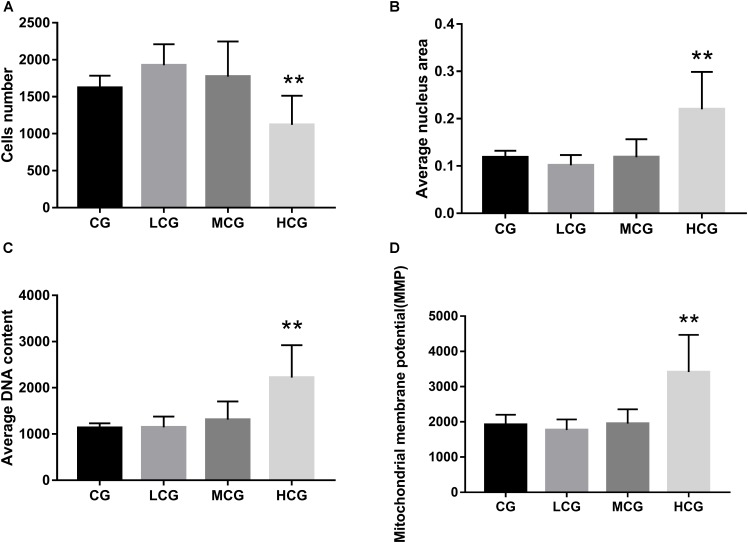
The cells number **(A)**, average nucleus area **(B)**, average DNA content **(C)** and MMP **(D)** of the four groups. ANOVA was used to calculate significant difference. ^∗^*P* < 0.05 and ^∗∗^*P* < 0.01, compared with the CG.

To further explore the effects of C-8-O-β-D-glu on L-02 cells, HCA was applied to examine the changes of nuclei and mitochondria after the cells being exposed to the drug for 24 h. The representative cell staining images of the four groups were shown in Figure 3, which showed an increase of cells number in LCG and a decrease in HCG when compared with CG. Based on staining on nuclei by Bisbenzimide H 33342, the number of L-02 cells was counted. The results were in accord with the MTT assay (Figure 4A) that HCG showed significant cytotoxicity compared with CG (*p* < 0.01). It was also observed that the average nucleus area and average DNA content of L-02 cells in HCG were significantly increased (Figures 4B,C) (*p* < 0.01). As for mitochondria, the MMP was calculated from the intensity of Rho123 staining divided by cells number. As shown in Figure 4D, the MMP of HCG was significantly higher than that of CG (*p* < 0.01). However, no statistical difference of cells number, average nucleus area, average DNA content and MMP can be found among LCG, MCG and CG. These results above suggested that the hepatotoxic mechanisms of C-8-O-β-D-glu at high concentration might be related to the changes in nuclei and mitochondria.

### UPLC-MS/MS Fingerprinting

**FIGURE 5:**
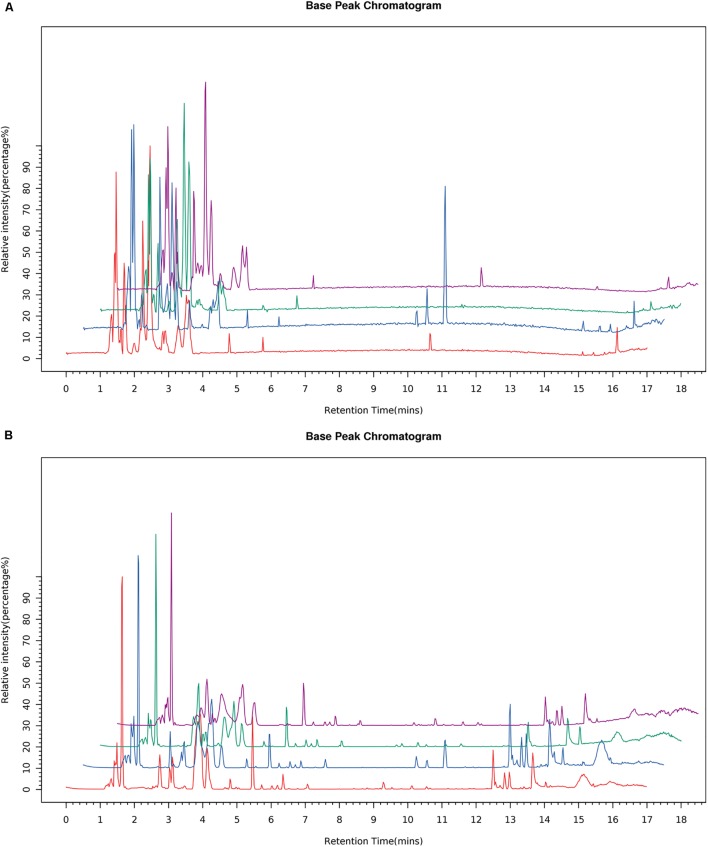
Typical BPC of the cells sample from the four groups in the ESI negative **(A)** and positive ion modes **(B)**.

All cell samples were analyzed by UPLC-MS/MS in both positive and negative ionization modes, and the representative BPC (Figure 5), of four groups were obtained under the optimal conditions. The chromatographic peaks considered to be the representative chemical fingerprints of endogenous metabolites were detected within 18 min with some remarkable differences observed among the four groups.

### Multivariate Statistical Analysis

**FIGURE 6:**
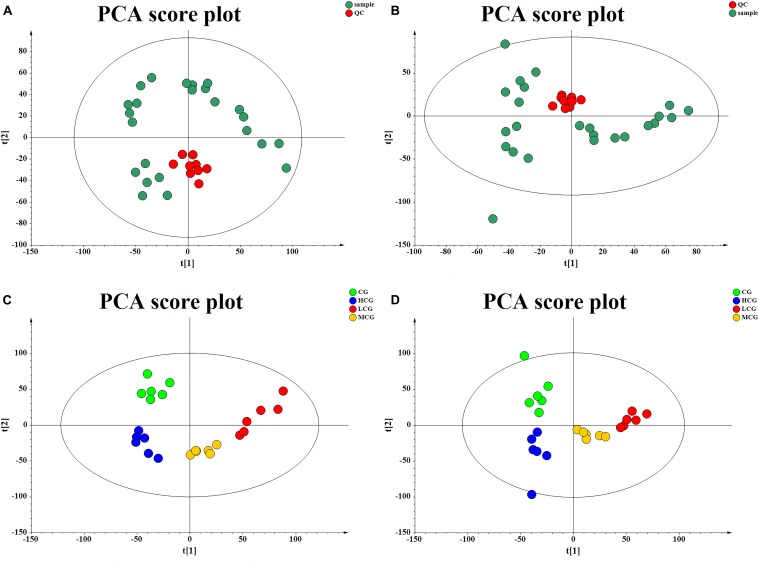
The score plot of QC samples and test samples from PCA in negative mode **(A)** and positive mode **(B)**, respectively. The score plot of the CG, LCG, MCG, and HCG from PCA in negative mode **(C)** and positive mode **(D)**.

The QC samples were used to correct the deviations of analysis results from mixed samples and the errors caused by analytical instrument. PCA score plots (Figures 6A,B) showed that the QC samples gathered together in both positive and negative mode, which indicated that the test samples and instrument were stable in long-term run.

**FIGURE 7:**
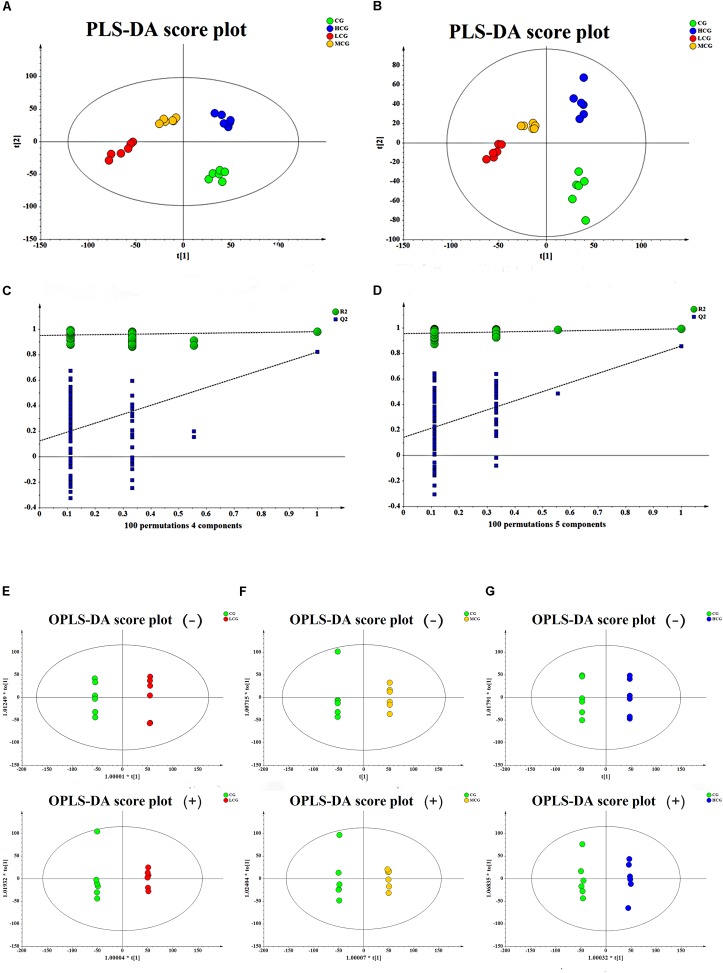
The score plot of the CG, LCG, MCG and HCG from PLS-DA in negative mode **(A)** and positive mode **(B)**, respectively. 100-permutation test of PLS-DA model in negative mode **(C)** and positive mode **(D)**. The score plot of CG vs. LCG **(E)**, CG vs. MCG **(F)**, CG vs. HCG **(G)** from OPLS-DA in negative mode and positive mode, respectively.

To obtain the difference of metabolic components among four group samples, multivariate statistical analysis method was used to screen the metabolites of each sample. PCA score plot (Figures 6C,D) showed a great separation trend of CG and drug experimental groups (R2X = 0.215, Q2 = 0.048 in negative mode; R2X = 0.223, Q2 = 0.025 in positive mode). Supervised analysis including PLS-DA and OPLS-DA was applied to identify the differences and outliers between CG and drug experimental groups. The cross test parameters R2X, R2Y, and Q2 values of PLS-DA model were 0.312, 0.989, and 0.805 in negative mode and 0.371, 0.994, and 0.841 in positive mode, which suggested that fitness and prediction of the model were good. Moreover, the permutation test was applied to evaluate whether the model was overfitting. The results shown in Figures 7C,D indicated that the model was valid. As shown in PLS-DA score plot (Figures 7A,B), every group was clearly separated from each other in both positive and negative modes, which indicated that there were significant metabolite differences among the groups. It also suggested that the effects of C-8-O-β-D-glu on L-02 cells were related to the concentration. OPLS-DA was carried out to further confirm the potential differential metabolites of drug experimental groups compared with CG, respectively. The OPLS-DA score plot was shown in Figures 7E–G. The VIP values of the metabolites were calculated based on this to screen the potential biomarkers.

### Screening and Identification of the Potential Biomarkers, and Metabolic Pathway Analysis

**FIGURE 8:**
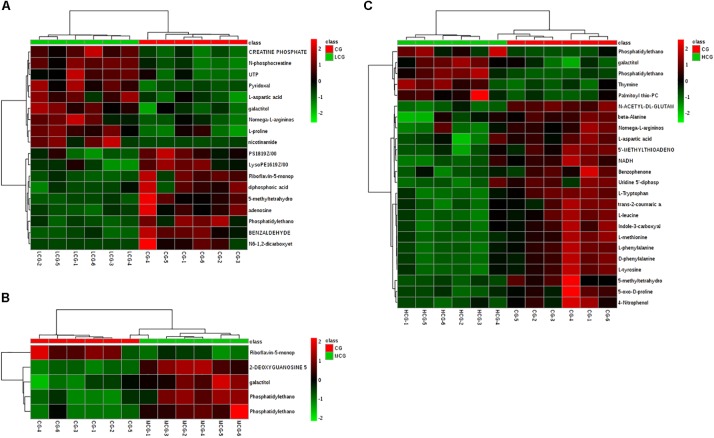
Heat-map of differential metabolites of CG compared with LCG **(A)**, MCG **(B)**, and HCG **(C)**, respectively. Rows: differential metabolites; columns: samples. The color of each small square represents the level of metabolite expression. Red: highest; green: lowest; black; mean.

VIP values reflected the influence of each variable, and a larger distance indicated a more important projection. Therefore, all the metabolites were selected according to the VIP values from OPLS-DA firstly. The corrected *p*-values (*q*-values) between CG and drug experimental groups were also applied to screen the differential metabolites. Ultimately, 42 endogenous metabolites contributing most to the separation of drug experimental groups from CG were selected, which might account for the effects of C-8-O-β-D-glu on L-02 cells. These metabolites were identified according to MS/MS information combined with online database information. There were 18 and 5 identified differential metabolites between CG and LCG or MCG, respectively. As for biomarkers of hepatotoxicity of C-8-O-β-D-glu, 26 metabolites were identified as differential metabolites between HCG and CG. Changes of all the differential metabolites between CG and drug experimental groups were shown in Figure 8, a heat map.

**FIGURE 9:**
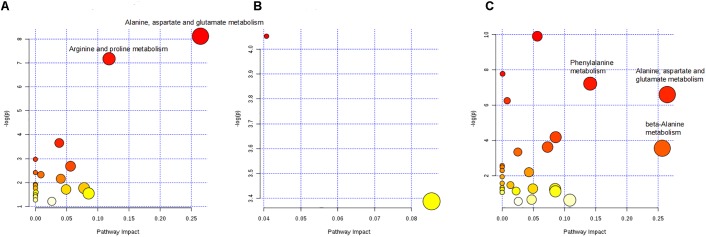
Pathway analysis on differential metabolites of LCG vs. CG **(A)**, MCG vs. CG **(B)**, and HCG vs. CG **(C)**. The circles marked with name of the pathways were the identified important pathways.

The pathways influenced by the changes of above metabolites were identified by metabolic pathway enrichment and topology analysis through MetPA database (Figure 9). The -log(p) value from the pathway enrichment analysis and the pathway impact value from the pathway topology analysis were calculated by MetaboAnalyst 4.0. The higher the -log(p) value and the pathway impact value, the more important the pathway. Four crucial pathways of C-8-O-β-D-glu on L-02 cells were finally identified based on the pathway impact and -log(p) value, which were summarized in Table 2 suggesting that different concentrations of C-8-O-β-D-glu would affect L-02 cells viability through the impacts on the pathways of alanine, aspartate and glutamate metabolism, arginine and proline metabolism, phenylalanine metabolism, beta-alanine metabolism in different ways.

**Table 2:** Metabolic pathways associated to varied influences of chrysophanol-8-O-β-D-glucoside on L-02 cell.

Metabolic pathways	Groups	Total	Hits	-LOG(p)	Impact
Alanine, aspartate, and glutamate metabolism	CG vs. LCG	24	3	8.1277	0.26496
	CG vs. HCG		3	6.6046	0.26496
Arginine and proline metabolism	CG vs. LCG	77	4	7.1824	0.1182
Phenylalanine metabolism	CG vs. HCG	45	4	7.2241	0.14126
beta-Alanine metabolism	CG vs. HCG	28	2	3.5614	0.25694


**FIGURE 10:**
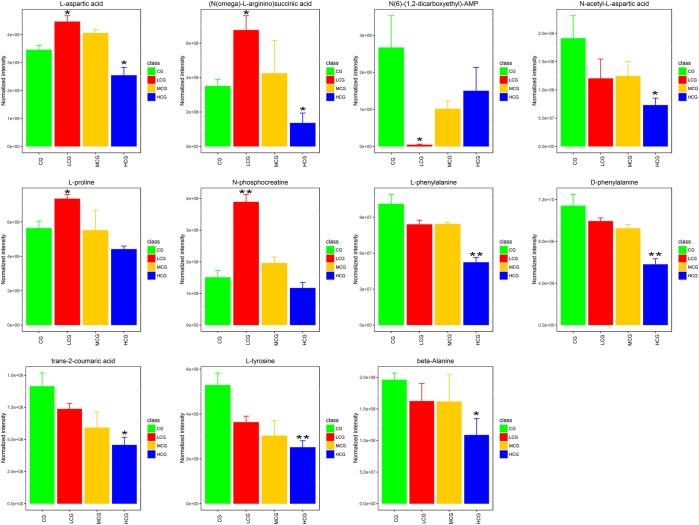
Relative intensities of the 11 important differential metabolites. ^∗^*q* < 0.05 and ^∗∗^*q* < 0.01, significant differences compared to the control group.

Remarked dysregulation of alanine, aspartate and glutamate metabolism were observed in both LCG and HCG. In addition, low concentration of C-8-O-β-D-glu would impact arginine and proline metabolism. High concentration of C-8-O-β-D-glu would impact phenylalanine metabolism and beta-alanine metabolism. There was no important pathway involved in MCG vs. CG. Based on these metabolic pathways, relevant important differential metabolites were selected and summarized in Table 3. As can be seen in Figure 10, relative intensities of most differential metabolites varied in a dose-dependent manner. For example, contents of L-aspartic acid, (N(omega)-L-arginino)succinic acid, L-proline, N-phosphocreatine, trans-2-coumaric acid and L-tyrosine decreased with increasing dose, while the content of N(6)-(1,2-dicarboxyethyl)-AMP increased with increasing dose.

**Table 3:** Identification results and change trends of important differential metabolites.

No.	Deduced metabolites	Elemental composition	mz	rtmin	VIP	*p*-value	*q*-value	KEGG	ESI mode	Related pathway	Change Trend		
											
											LCG	MCG	HCG
1	L-aspartic acid	C4H7NO4	132.03	1.41	1.53	0.000	0.000	C00049	–	Alanine, aspartate and glutamate metabolism, beta-Alanine metabolism, Arginine and proline metabolism	↑	↑	↓
2	(N(omega)-L-arginino)succinic acid	C10H18N4O6	291.13	1.50	1.09	0.019	0.030	C03406	+	Alanine, aspartate and glutamate metabolism, Arginine and proline metabolism	↑	–	↓
3	N(6)-(1,2-dicarboxyethyl)-AMP	C14H18N5O11P	462.07	4.94	1.10	0.021	0.032	C03794	–	Alanine, aspartate and glutamate metabolism	↓	–	–
4	N-acetyl-L-aspartic acid	C6H9NO5	176.05	2.59	1.56	0.018	0.048	C01042	+		–	–	↓
5	L-proline	C5H9NO2	116.07	1.66	1.08	0.031	0.040	C00148	+	Arginine and proline metabolism	↑	–	↓
6	N-phosphocreatine	C4H10N3O5P	210.03	1.50	1.58	0.000	0.000	C02305	–	Arginine and proline metabolism	↑	–	–
7	L-phenylalanine	C9H11NO2	164.07	5.47	1.86	0.000	0.000	C00079	–	Phenylalanine metabolism	–	–	↓
8	D-phenylalanine	C9H11NO2	166.09	5.47	1.60	0.000	0.000	C02265	+		–	–	↓
9	trans-2-coumaric acid	C9H8O3	165.05	3.76	1.27	0.009	0.018	C01772	+		–	–	↓
10	L-tyrosine	C9H11NO3	182.08	3.76	1.36	0.003	0.008	C00082	+	Phenylalanine metabolism	↓	–	↓
11	beta-Alanine	C3H7NO2	90.06	1.45	1.64	0.011	0.039	C00099	+	beta-Alanine metabolism	–	–	↓


*The shown change trends have significant differences in comparison with CG.*

## Discussion

A lot of studies about free anthraquinones inducing liver injury have been published. But little attention has been put on the effect of glycosylated anthraquinones on liver. In pre-screening experiment, we found that the effects of C-8-O-β-D-glu on liver cells viability were opposite with different concentrations. Thus, in this study, we performed the metabonomics and HCA research to further explore its mechanism. Before the experiments, the contents of C-8-O-β-D-glu in cell culture medium at different time were tested to ensure the stability during the whole experiment process.

### Different Concentrations of C-8-O-β-D-Glu Exposure Impacted L-02 Cells Viability and Cell State

Because of the poor solubility of C-8-O-β-D-glu, the concentration wasn’t high enough to detect the IC_50_ of C-8-O-β-D-glu. So the concentration with maximum solubility (96 μM) was used as the high dosage in MTT assay.

The results of MTT assay suggested that C-8-O-β-D-glu in low concentrations (12 and 24 μM) increased L-02 cells viability. However, HCA results didn’t show statistical difference in LCG and MCG compared to CG. It suggested that C-8-O-β-D-glu at lower concentrations did not affect the cells number or cells status of the nuclei and mitochondria of L-02 cells, which was inconsistent with MTT assay. Although the results of MTT assay implied a rise of mitochondrial activity in LCG, MMP analyzed by HCA remained unchanged. It indicated the mitochondria in LCG were not significantly affected. Since there was an upward trend in cells number of LCG (1925 ± 283 in LCG, 1594 ± 163 in CG) measured by HCA, the difference between the cells viability and cells number might be caused by the difference between the two experimental methods. The results of MTT assay and HCA experiment both indicated that high concentration (96 μM) of C-8-O-β-D-glu inhibited cells growth. Moreover, significant changes in cell nuclei and mitochondria were found in HCG. Normally, the cells injured by toxic substances showed nucleus shrinkage caused by cell apoptosis and necrosis. However, both the average nucleus area and DNA content were increased in HCG which needed further study. Normal MMP is mainly dependent on the proton pump located in the gap between mitochondrial matrix and mitochondrial membrane so that different protons between the matrix and the membrane maintain a certain electrochemical gradient. MMP reflects the properties of electron transport chain and changes in pathological conditions. Respiratory chain of mitochondria is the main site of ROS production (Brand and Nicholls, 2011). Under normal condition, mitochondria produce a small amount of ROS to maintain the normal physiological functions of cells. However, the reduced proton reflux increases MMP in pathological condition. Then electrons are leaked which resulted in producing a large number of ROS (Su et al., 2016). Finally, excessive ROS causes oxidative damage to cells (Ray et al., 2012). Thus, we conjectured that the increase of MMP level in HCG resulted in L-02 cells damage by affecting the production of ROS.

### Cell Metabolism Involved in Varied Influences of C-8-O-β-D-Glu With Different Concentrations on L-02 Cells

Changes of intracellular metabolites reflect abnormalities of cells’ physiological state. The extent of important metabolites change has a relationship with C-8-O-β-D-glu concentration. More importantly, the contents of several metabolites in LCG and HCG changed in different trends compared to CG, including L-aspartic acid, (N(omega)-L-arginino)succinic acid and L-proline. These metabolites might be more relevant to the different effects of C-8-O-β-D-glu on L-02 cells.

**FIGURE 11:**
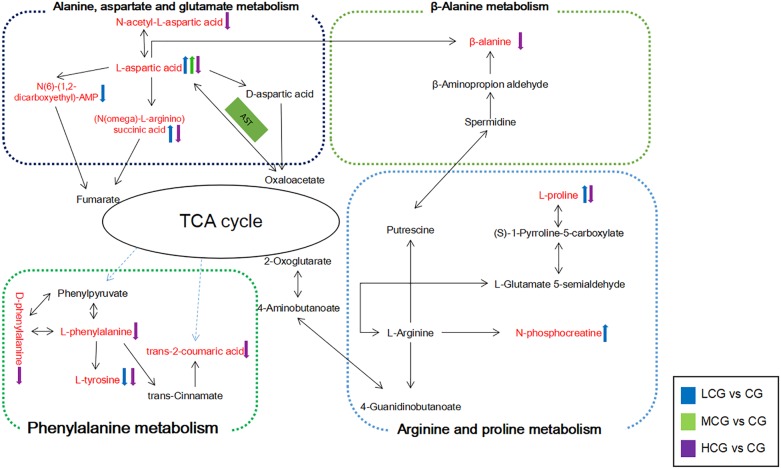
Schematic diagram of the metabolic pathway related to the effects of different concentrations of C-8-O-β-D-glu on L-02 cells. The metabolites in red represented the potential biomarkers identified in this research. The arrows next to the metabolites represented their change trends in each drug group. The substances in the green box represented the enzymes needed for the reaction.

In present research, three concentrations (24, 48, and 96 μM) of C-8-O-β-D-glu affected different metabolic pathways in varying degrees to induce different effects on L-02 cells viability. The schematic diagram of these pathways was shown in Figure 11. The metabolism of amino acids occurs mainly in liver. Therefore, physiological changes of liver are usually accompanied with the abnormality of amino acid metabolism. Previous metabonomics studies have shown that anthraquinones would induce liver injury by disordering amino acid metabolism, including tryptophan metabolism (Zhang et al., 2016), phenylalanine metabolism, alanine, aspartate and glutamate metabolism (Li et al., 2017), L-threonine and serine metabolism (Zhang et al., 2015). In this research, we found amino acid metabolism was involved in most identified pathways.

Low concentration of C-8-O-β-D-glu might promote L-02 cells viability by regulating the expression of alanine, aspartate and glutamate metabolism, arginine and proline metabolism. High concentration of C-8-O-β-D-glu also affected alanine, aspartate and glutamate metabolism, but to the contrary trend. Thus, differential metabolites including L-aspartic acid, (N(omega)-L-arginino)succinic acid, N(6)-(1,2-dicarboxyethyl)-AMP and N-acetyl-L-aspartic acid could be considered as potential biomarkers of C-8-O-β-D-glu affecting liver cells viability. In addition, arginine and proline metabolism was significantly changed in LCG. High concentrations of C-8-O-β-D-glu disrupted phenylalanine metabolism and beta-alanine metabolism. The results indicated that the mentioned metabolites were closely related to promoting or toxic effects of C-8-O-β-D-glu. In MCG, there was no significant change in cellular metabolic pathway. This was consistent with MTT results that medium concentration had little effect on cell viability.

L-aspartic acid is an important non-essential amino acid which participates in many biochemical processes including urea cycle, gluconeogenesis, and malate-aspartate shuttle etc. Rising content of L-aspartic acid in LCG indicated a gain in energy generation via these processes. N-phosphocreatine and L-proline were also up-regulated in LCG. N-phosphocreatine is an important energy source for cells. Promotion of these processes related to energy generation will result in an increase in L-02 cells viability.

The content of L-aspartic acid, as well as its by-products (N(omega)-L-arginino)succinic acid and N-acetyl-L-aspartic acid were decreased in HCG. One important physiological function of L-aspartic acid is providing an ammonia molecule for the urea cycle to remove excess ammonia (Mew et al., 2011). Thus, the decrease in L-aspartic acid content will cause ammonia accumulation and damage to the liver. (N(omega)-L-arginino)succinic acid is synthesized from L-aspartic acid by the loss of ammonia. The decline of (N(omega)-L-arginino)succinic acid in HCG conformed to the decrease of L-aspartic acid which can be produced from oxaloacetate via transamination by the function of aspartate aminotransferase (AST). AST is also one of the most commonly used indicators for diagnosis of liver disease. When the hepatocyte is damaged, the permeability of the cell membrane increases, and the AST outflows from the cell (Otto-Ślusarczyk et al., 2016). The decrease in L-aspartic might be caused by the outflow of AST. Based on these facts, we speculated that high concentration of C-8-O-β-D-glu induced L-02 cells damage via reducing AST in cells, inhibiting the production of L-aspartic acid, increasing the amount of ammonia and finally aggravating the liver injury. In addition, L-proline in HCG was significantly reduced. It has been reported that L-proline can protect cells by directly cleaning up ROS, protecting and up-regulating the antioxidant enzymes, and maintaining the key redox molecules (Krishnan et al., 2008; Szabados and Savouré, 2010; Natarajan et al., 2012). According to our previous speculation about the result of HCA, high concentration of C-8-O-β-D-glu induced L-02 cells damage might be related to oxidative stress. Therefore, the decreasing of L-proline probably aggravated the damage caused by oxidative stress.

This study revealed that the different effects of C-8-O-β-D-glu on liver L-02 cell with varying concentrations, which prompts us to pay more attention to dosage applied in clinic. But the dosage-relationship of effect or toxicity remained obsure which will be investigated in the further experiment.

## Conclusion

This study explored the different effects of three concentrations of C-8-O-β-D-glu on L-02 liver cells that high concentration induced L-02 cells damage, while low concentration promoted the cells viability. The HCA results indicated that high concentration of C-8-O-β-D-glu can significantly reduce the cells number, increase average nucleus area, DNA content and MMP. The results of metabonomics analysis indicated that metabolic profiles of each group were clearly separated. Eleven important differential metabolites associated with four pathways including alanine, aspartate and glutamate metabolism, arginine and proline metabolism, phenylalanine metabolism, pantothenate and beta-alanine metabolism were identified in this study. Based on these results, it is concluded that different concentrations of C-8-O-β-D-glu could impact L-02 cells via disturbing metabolism in different ways.

## Author Contributions

ML and YL conducted the experiments. ML and YL wrote the manuscript and prepared the figures. XG, YQ, and YZ conducted the sample collection and data analysis. CP conceived the study.

## Conflict of Interest Statement

The authors declare that the research was conducted in the absence of any commercial or financial relationships that could be construed as a potential conflict of interest.

## Footnotes

1 http://www.hmdb.ca

2 http://metlin.scripps.edu

3 http://www.massbank.jp/

4 http://www.lipidmaps.org

5 https://www.mzcloud.org

6 http://www.kegg.jp/

7 http://www.metaboanalyst.ca

## Abbreviations

BPC: Base peak chromatogram
C-8-O-β-D-glu: Chrysophanol-8-O-β-D-glucopyranoside
CG: Control group
DMEM: Dulbecco’s modified eagle medium
DMSO: Dimethyl sulfoxide
FBS: Fetal bovine serum
FDR: False discovery rate
HCA: High content analysis
HCG: High concentration group
IC50: 50% inhibitory concentration
LCG: Low concentration group
MCG: Medium concentration group
MMP: Mitochondrial membrane potential
MTT: 3-(4,5-dimethylthiazol-2-yl) 2,5- diphenyltetrazolium bromide
OPLS-DA: Orthogonal projection to latent structure discriminate analysis
PBS: Phosphate buffer saline
PCA: Principal component analysis
PLS-DA: Partial least squares discriminant analysis
Rho123: Rhodamine 123
ROS: Reactive oxygen species
UPLC-MS/MS: Ultra-performance liquid chromatography – mass spectrometry/mass spectrometry
VIP: Variable importance in the projection
